# Kinetic Study of Subcritical Water Extraction of Scopoletin, Alizarin, and Rutin from *Morinda citrifolia*

**DOI:** 10.3390/foods10102260

**Published:** 2021-09-24

**Authors:** Roslina Jamaludin, Dong-Shin Kim, Liza Md Salleh, Sang-Bin Lim

**Affiliations:** 1 Centre of Lipids Engineering & Applied Research (CLEAR), Ibnu Sina Institute for Scientific and Industrial Research, Universiti Teknologi Malaysia, UTM, Johor Bahru 81310, Johor, Malaysia; roslina_9@yahoo.com (R.J.); r-liza@utm.my (L.M.S.); 2 Department of Bioprocess and Polymer Engineering, School of Chemical and Energy Engineering, Faculty of Engineering, Universiti Teknologi Malaysia, UTM, Johor Bahru 81310, Johor, Malaysia; 3 Department of Food Bioengineering, Jeju National University, Jeju 63243, Korea; feel567@naver.com

**Keywords:** *Morinda citrifolia*, subcritical water extraction, scopoletin, alizarin, rutin, kinetic study

## Abstract

Noni fruits (*Morinda citrifolia*) are a source of phenolic bioactive compounds (scopoletin, alizarin, and rutin), which have antioxidant, antimicrobial, anticancer, and anti-inflammatory activities. In this study, subcritical water was applied to determine the extraction yields and kinetics of phenolic compounds from noni fruits. The scopoletin and alizarin yields increased with the increase in temperature from 100 to 140 °C, while that of rutin increased up to 120 °C and then decreased at 140 °C. The yields of all the compounds rapidly increased from 1 to 2 mL/min and then slightly up to 3 mL/min of water flow rate. The extraction kinetics were assessed using two mathematical models. The two-site kinetic desorption model had a better fit for all experimental conditions throughout the extraction cycle and best described the extraction kinetics of phenolic compounds from noni fruits. The diffusion coefficients of scopoletin and alizarin at 140 °C and 3 mL/min were 3.7- and 16.2-fold higher than those at 100 °C and 1 mL/min, respectively. The activation energies of alizarin were 2.9- to 8.5-fold higher than those of scopoletin at various flow rates. Thus, subcritical water could be an excellent solvent with higher extraction yields and shorter extraction times using an environmentally friendly solvent.

## 1. Introduction

Noni (*Morinda citrifolia*), also known as mengkudu in Malaysia, which belongs to the Rubiaceae family, is native to Southeast Asia and has been cultivated and naturalized across Australia and the Pacific Islands. Noni has been used as traditional Polynesian medicines and is often consumed to treat high blood pressure, stimulate the immune system, and treat bacterial, viral, fungal infections and many more. Its ability to treat various illnesses has led numerous scientific studies to be carried out to investigate phytochemicals and their pharmacological activities from different parts of noni [1,2,3].

Noni contains various phytochemicals that are important for foods, supplements, and nutraceuticals. About 200 phytochemicals have been identified in noni, and the major groups of functional components are phenolics, alkaloids, and organic acids. Among them, the most important are scopoletin (phenolic coumarin), alizarin, rutin, damnacanthal, morindone, morindin, aucubin, asperulose, rubiadin, and anthraquinone glycosides [4,5,6,7].

Scopoletin is one of the most important coumarins in noni fruits. It possesses a variety of pharmacological effects, such as ameliorated hyperglycemia and hepatic steatosis [8], and anti-inflammatory activity [9]. Alizarin is found mainly in noni roots with small traces in fruits and leaves. It has an antiangiogenic effect by blocking blood circulation to malignant tumors and could be a potential antitumor drug for bone cancer [6,10,11]. Another interesting phenolic compound is rutin, which possesses antiadrenergic and antidopaminergic activities [12] and diabetic cardiomyopathy characteristics [13].

Selecting the suitable extraction method is a crucial step to produce high yields of phenolic compounds from noni fruits for functional foods and nutraceuticals. There are green processing technologies that can be applied to extract phenolic compounds from biomaterials, such as supercritical fluid, microwave-assisted, subcritical water (SW), and hydrostatic pressure extractions [14,15,16]. Increased interests have been recorded in the use of SW for the extraction of phenolic compounds from plant or various biomass materials due to its eco-friendly method by using only water as main solvent. SW typically refers to the state of liquid water retained by pressure beyond the boiling point (100 °C), up to the state of its critical temperature (374 °C). The occurring breakdown of the intermolecular hydrogen bonds due to the subcritical state causes a corresponding decrease in polarity of the water. The interesting point of SW is that the dielectric constant, viscosity, and surface tension of water can be adjusted by applying different operational temperatures. Therefore, SW can become an effective extraction solvent for medium polar and nonpolar compounds [15,17,18,19,20,21].

During SW extraction (SWE), the bound solute inside the cells is extracted from the sample matrix, and then is drained to the solvent [22,23]. Investigating the extraction mechanisms based on mathematical kinetic models can help understand the extraction process [24,25,26]. This will provide useful information for scale-up and designing of new commercial subcritical water extraction devices [22,25].

Several research studies have been performed on the modeling of SWE kinetics for: damnacanthal from roots of *M. citrifolia* [25], carbohydrates and phenolics from flax shives [22], anthraquinones from *Heterophyllaea pustulata* [26], polyphenols from grape by-products [27], rutin from *Fagopyrum esculentum* [28], and bioactive compounds from malted quinoa [29]. To the best of our knowledge, there have been no research studies reported on the extraction kinetic modeling of scopoletin, alizarin, and rutin from noni fruits using SW.

In this study, the effect of operating parameters (water temperature and flow rate) using SW on the recovery and extraction kinetics of phenolic bioactive compounds from noni fruits was investigated. Two mathematical kinetic models (two-site kinetic and partition coefficient models) were compared to investigate the mechanism controlling the extraction rates using SW.

## 2. Materials and Methods

### 2.1. Sample Material and Chemicals

Noni fruits were obtained from Johor, Malaysia; washed using tap water; sliced to about 5 mm; and freeze-dried overnight until a constant weight was achieved for 32 h. The dried whole fruits with seeds and skins (moisture content: 5.82 ± 0.06%) were then ground using a Waring blender (mean particle size of 575 µm). Scopoletin (≥99%) and alizarin (97%) were purchased from Sigma Chemical Co. (St. Louis, MO, USA). Rutin (≥95%) was purchased from Santa Cruz Biotechnology, Inc. (Santa Cruz, CA, USA). High pressure liquid chromatography (HPLC)-grade acetonitrile (99.8%), methyl alcohol (99.9%), and formic acid (99%) were obtained from Sigma Chemical Co.

### 2.2. Methanol Extraction

To determine the amounts of scopoletin, rutin, and alizarin in the dried raw sample, solvent extraction with methanol was carried out. The dried noni fruits (0.5 g) were added to methanol (20 mL) and stirred for 30 min. The mixture was centrifuged (3000× *g*, 10 min) to separate the supernatant, and the residue was extracted four more times until all three compounds were quantitatively recovered. The extract was evaporated using a vacuum rotary evaporator, redissolved in methanol, and filtered using a syringe filter (0.45 μm) for HPLC analysis. The analysis data of phenolic compounds were averaged with three replicates.

### 2.3. Subcritical Water Extraction

**Figure 1:**
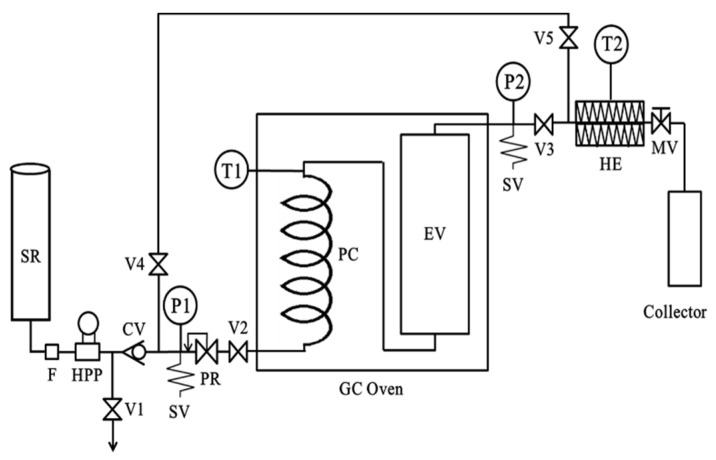
Semicontinuous subcritical water extraction system (CV, check valve; EV, extraction vessel; F, filter; HE, heat exchanger; HPP, high-pressure pump; MV, metering valve; *p*, pressure gauge; PC, preheating coil; PR, pressure regulator; SV, safety valve; SR, solvent reservoir; T, temperature indicator; V, on/off valve).

SWE was carried out in our custom-made semicontinuous extractor (Figure 1) described previously by Kim and Lim [28]. The extraction method is briefly described here. The freeze-dried noni fruit powder (2 g) was mixed with sea sand and loaded in the stainless steel extraction cell (7.8 mm i.d. × 300 mm length). Glass wool was inserted at both ends of the extractor to prevent plugging. The extraction process was commenced by switching on the oven as soon as the temperature set point was reached. The degassed water was supplied by a piston pump (Thermo Separation Products, Waltham, MA, USA). To retain the liquid state of the water at high temperature, the pressure of the water was maintained at 5 MPa. The water flow rate was controlled by a high-pressure metering valve (Parker Autoclave Engineers, Erie, PA, USA). The extracts were taken every 2.5 min for 20 min. The water temperatures studied in this work were 100, 120, and 140 °C with water flow rates of 1, 2, and 3 mL/min based on our preliminary experiments. All the experiments were repeated three times.

### 2.4. HPLC Analysis

The contents of phenolic compounds in the extracts were measured by HPLC (Alliance 2965, Waters Corp., Milford, MA, USA) described previously by Kim and Lim [28]. The analysis method is briefly described here. The phenolic compounds were separated on an XTerra^®^ C18 column (250 × 4.6 mm, 5 µm film thickness; Waters Corp, Milford, MA, USA). The mobile phases consisted of 0.5% formic acid (A) and acetonitrile (B). It was initially set at 80% A and 20% B, and then the ratio of solvent B was changed as follows: to 20% over 15 min, to 70% over 20 min, and to 20% over 30 min. The mobile phase flow rate was 1.0 mL/min. The detection of scopoletin and rutin was performed at 350 nm, while that of alizarin at 250 nm using a photodiode array detector (Alliance 2998, Waters Corp.). Quantification was performed by comparing the chromatographic peak areas with those of external standards (Figures S1–S3).

### 2.5. Kinetic Modeling

To describe the extraction kinetics of phenolic compounds under subcritical conditions, two kinetic models were applied. The partition coefficient model assumes that the thermodynamic equilibrium mainly controls the extraction process [30]. The extraction curve for the partition coefficient model is given in Equation (1):

(1) $$\frac{S_{b}}{S_{0}}=\frac{S_{a}}{S_{0}}+\frac{1-S_{a}{/S}_{0}}{{1+K}_{D}m/\rho \left(V_{b}-V_{a}\right)}$$

where *S*_0_ is the initial solute (mg/g) in the raw noni fruits; *S_a_* and *S_b_* are the extracted solutes (mg/g) using water *V_a_* and *V_b_* (mL), respectively; *m* and *ρ* are the sample (mg) and density of water (mg/mL) under given temperatures; and *K_D_* is the equilibrium partitioning coefficient. The model parameters were determined using the Microsoft Excel software.

The two-site kinetic model considers the extraction controlled by an internal diffusion. Two types of rate constants, *k*_1_ (for the fast-released fraction, *f*) and *k*_2_ (for the slowly released fraction, 1 − *f*), are used to describe the extraction curve [27]. The mathematical expression for calculating the total mass of solute recovered after time t is as follows:

(2) $$\frac{S_{t}}{S_{0}}=1-\left(f\exp\left(-k_{1}t\right)\right)-\left[\left(1-f\right)\;\exp\left(-k_{2}t\right)\right]$$

where *S*_0_ is the initial solute (mg/g) in the raw noni fruits and *S*_t_ is the recovered solute (mg/g) after a certain time (min). The model parameters were determined using Matlab curve-fitting function.

## 3. Results and Discussion

### 3.1. Effect of Subcritical Water Temperature

**Figure 2:**
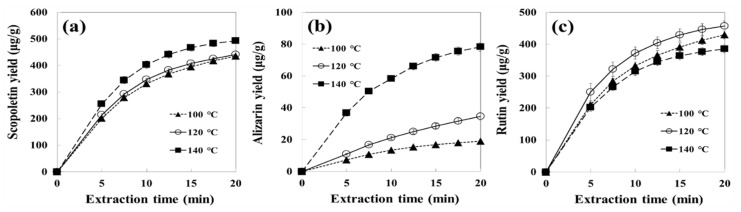
Effect of three different water temperatures on cumulative yields of (**a**) scopoletin, (**b**) alizarin, and (**c**) rutin under a water flow rate of 2 mL/min from noni fruits extracted by subcritical water. Data are mean values ± SD (*n* = 3).

In SWE, the extraction temperature plays an important role that affects the dielectric constant, viscosity, and surface tension of water, while pressure has little effect as long as the water remains liquid in the system [24,31,32]. In this study, the extraction yields of phenolic compounds were determined at 100–140 °C and 2 mL/min (Figure 2). The extraction (or recovery) yields (%) of phenolic compounds were calculated based on the contents in the raw noni fruits (scopoletin: 533.4, rutin: 544.9, and alizarin: 23.4 µg/g dry matter). The scopoletin yield slightly increased at 120 °C, but significantly at 140 °C compared with 100 °C. The alizarin yields gradually increased as the temperature increased from 100 to 120 °C, but rapidly increased at 140 °C. The yield of rutin increased as the temperature increased from 100 to 120 °C, but at 140 °C, it decreased due to thermal degradation. Kim and Lim [28] also experienced the degradation of rutin into low-molecular-weight compounds at over 120 °C of water.

The scopoletin yields were 435.3, 441.3, and 493.3 µg/g dry sample (81.6%, 82.7%, and 92.4% compared with the content in the raw sample) at 100, 120, and 140 °C, respectively (Table 1). Tatke and Rajan [33] reported that the yields of scopoletin were 7.9%, 9.5%, 10.9%, 31.8%, and 45.1% for supercritical fluid, Soxhlet, reflux, ultrasonic-assisted, and microwave-assisted extractions from *Convolvulus pluricaulis*, respectively.

The alizarin yields were 19.1, 34.5, and 78.3 µg/g dry sample (81.6%, 147.4%, and 334.6% compared with the content in the raw sample) at 100, 120, and 140 °C, respectively. The alizarin contents of the extracts at 120 and 140 °C were higher than that contained in the original raw sample probably due to the hydrolysis of alizarin glycosides to their corresponding aglycones at high water temperature [34,35]. Alizarin is known to exist as free and glycoside forms, and the glycoside-to-aglycone ratio is known to be 237:1 mg/mL in ethanol extract from the roots of Iranian madder [34,36,37]. The rutin yields were 429.6, 457.0, and 385.2 µg/g dry sample (78.8%, 83.8%, and 70.6% compared with the content in the raw sample) at 100, 120, and 140 °C, respectively.

The increase in extraction yield with water temperature is due to the decrease in dielectric constant, viscosity, and surface tension of water at high temperatures [18,25]. The dielectric constants of water at 20, 100, 120, and 140 °C were 80.2, 55.4, 50.5, and 46.0, respectively [38]. The viscosities of water at 20, 100, 120, and 140 °C were 1002 × 10^−6^, 281 × 10^−6^, 232 × 10^−6^, and 196 × 10^−6^ Pa·s, respectively [39]. The surface tensions of water at 20, 110, 130, and 150 °C were 72.5, 56.9, 52.7, and 48.3 mN/m, respectively [38]. This unique property is an advantage to SW, where it can extract medium polar compounds using water as a green solvent.

### 3.2. Effect of Water Flow Rate

**Figure 3:**
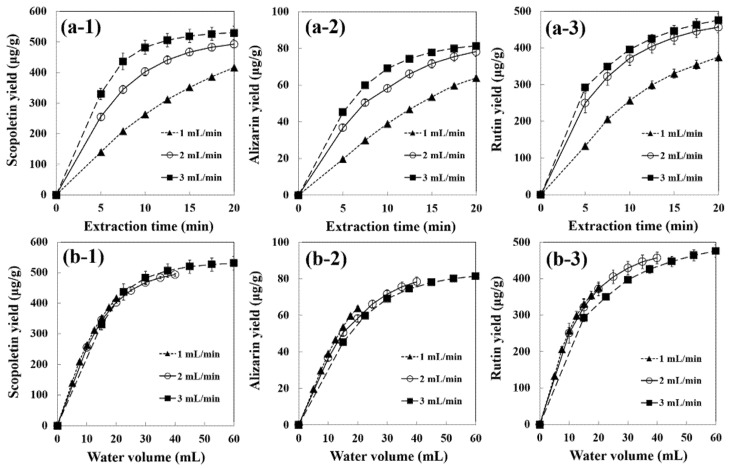
Effect of water flow rate (**a**) and water volume (**b**) on cumulative yields of scopoletin (**a1**,**b1**) at 140 °C, alizarin (**a2**,**b2**) at 140 °C, and rutin (**a3**,**b3**) at 120 °C from noni fruits extracted by SWE. Data are mean ± SD (*n* = 3).

The effect of flow rate on the extraction yields of phenolic compounds from noni fruits was also investigated in the range of 1 to 3 mL/min at 140 °C for scopoletin and alizarin, and at 120 °C for rutin. The changes of the extraction rate with the water flow rate and water volume can give an idea on the extraction kinetics [22,24,25,26,40]. The yield significantly increased with the increase in flow rate from 1 to 2 mL/min, but slightly increased at 3 mL/min (Figure 3a). The extraction curves were plotted against the water volume used, and all extraction curves were overlapped (*p* < 0.05) (Figure 3b), which indicates that the yields of phenolic compounds were directly proportional to the volume of water passing through the sample. Therefore, the extraction process of phenolic compounds from noni fruits using SW seems to be controlled by external mass transfer.

The scopoletin yields were 416.4, 493.3, and 530.6 µg/g dry sample (78.0%, 92.4%, and 99.4% compared with the content in the raw sample) at 140 °C and 1, 2, and 3 mL/min, respectively. The yield of scopoletin by SWE at 140 °C and 3 mL/min for 20 min using 60 mL of water was 530.6 µg/g dry sample compared with 533.4 µg/g dry sample using 100 mL of neat methanol for 150 min at ambient temperature. Therefore, SWE has the advantage of using an eco-friendly solvent instead of organic solvents, a small amount of solvent, and a shortened extraction time. The alizarin yields were 64.0, 78.3, and 81.5 µg/g dry sample (273%, 334%, and 348%) at 140 °C and 1, 2, and 3 mL/min, respectively. The yields of rutin were 375.5, 457.0, and 476.2 µg/g dry sample (68.9%, 83.8%, and 87.3%) at 120 °C and 1, 2, and 3 mL/min, respectively. During the extraction, increases in the concentration gradient of the solute between the sample matrix and water with an increasing flow rate lead to an increase in recovery yields [41].

### 3.3. Kinetic Modeling

The extraction mechanism is important to understand how the extraction works on a certain compound from plant materials. Generally, the extraction process can be explained by intraparticle diffusion or elution of the solute from a solid matrix into a flowing solvent [22,23]. The initial concentrations of scopoletin and rutin in the raw sample were set to C_0_ in the model equations. For alizarin, the maximum cumulative yield in the SW extract at 140 °C collected for 30 min was used as C_0_ in the model equations because the contents in the extracts were higher than those in the raw dried noni fruits due to the hydrolysis of alizarin glycosides to their corresponding aglycones at high water temperature.

The experimental data were fitted to the partition coefficient model, and the *K_D_* values of each compound were determined (Table 2). The *K_D_* values with different flow rates at the same temperature were the same (*p* < 0.05) and decreased as an increase in temperature from 100 to 140 °C. The decrease in *K_D_* value with an increase in water temperature indicates an increase in recovery efficiency due to weakened competition of the solute between the sample matrix and water at high temperature [23,30]. Alizarin has high *K_D_* values (9.1−74.2) compared with scopoletin (7.2−9.3) and rutin (6.5−9.3) at 100−140 °C. The *K_D_* value of alizarin was 8.2-fold higher compared with that of scopoletin at 100 °C. However, the *K_D_* value of alizarin decreased rapidly as the temperature increased to 140 °C to become comparable to that of scopoletin probably due to the acceleration of hydrolysis of alizarin glycosides to alizarin aglycone in water at 140 °C [34,35].

For the two-site kinetic model, two rate constants are calculated (Table S1). When the water temperature and flow rate were changed from 100 °C, 1 mL/min, to 140 °C, 3 mL/min, the *k*_1_ value for alizarin increased 18.3-fold compared with 3.88-fold for scopoletin and 3.56-fold for rutin. Likewise, the *f* value for alizarin also increased 3.92-fold compared with 1.93-fold for scopoletin and 1.43-fold for rutin. The desorption rate constants of the slowly released fraction (*k*_2_) for all compounds increased with increasing extraction temperature but decreased with increasing water flow rate (*p* < 0.05) due to the increased internal mass transfer resistance with increasing water flow rate [42].

**Figure 4:**
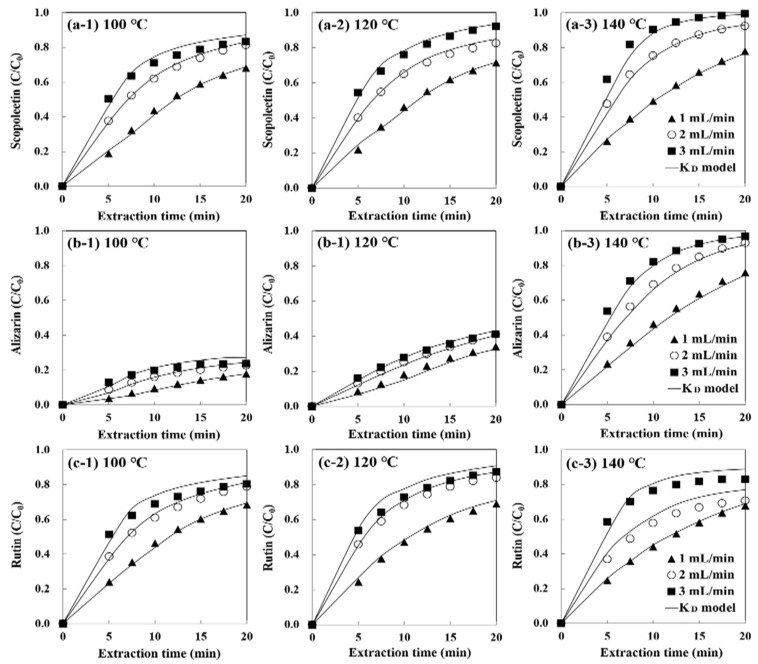
The thermodynamic partitioning model fit for the experimental data on subcritical water extraction of (**a**) scopoletin, (**b**) alizarin, and (**c**) rutin from noni fruits. The points represent the experimental values, and the solid lines are predicted in this model equation.

**Figure 5:**
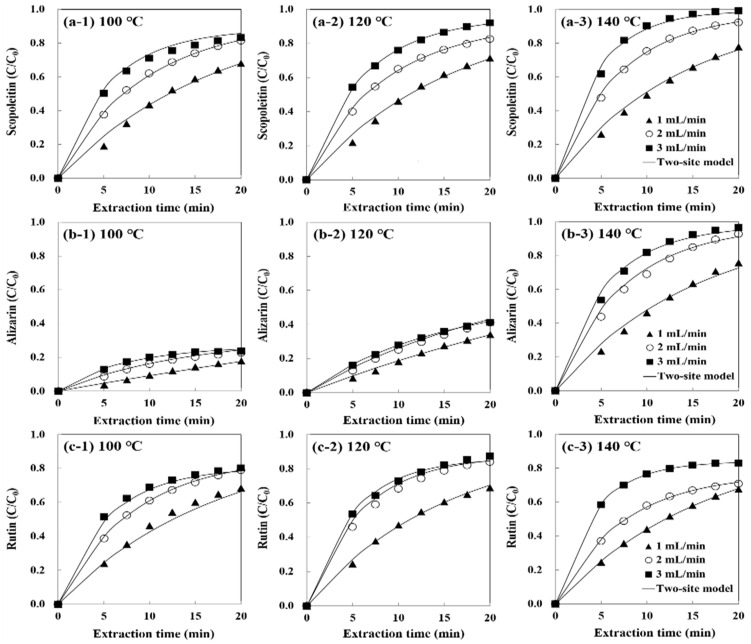
The two-site kinetic desorption model fit for the experimental values on subcritical water extraction of (**a**) scopoletin, (**b**) alizarin, and (**c**) rutin from noni fruits. The points represent the experimental values, and the solid lines are predicted in this model equation.

Figure 4 and Figure 5 show the partition coefficient, and two-site kinetic model fits with the extraction data of phenolic compounds from noni fruits. *The* *K_D_* model had no good fit in their latter parts at a higher flow rate (Figure 4). On the contrary, all regression curves by the two-site kinetic desorption model had a better fit for three phenolic compounds at all extraction conditions (Figure 5).

To quantitatively prove the suitability of two kinetic models, the correlation coefficient (*R*^2^) and the root mean square error (RMSE) between the values predicted by each model equation were calculated (Table S2). For the *K_D_* model, almost all the extraction conditions show a relatively low RMSE (≤0.047) and high *R*^2^ (≥0.967). Conversely, the two-site kinetic model shows the lowest RMSE (≤0.029) and highest *R*^2^ (≥0.991) for all temperature and flow rates. This indicates the better suitability of the two-site kinetic model in describing the extraction kinetics of phenolic compounds from noni fruits. Since this model takes into account two different solute fractions (i.e., fast and slow extraction periods) and divides the extraction curve into two desorption rate constants, *k*_1_ and *k*_2_, it enables a better representation of all phases of the extraction curve [27]. The regression curves of the two-site kinetic desorption model for rutin were suitable for degradation temperature (140 °C) and extraction temperatures (100 and 120 °C). This is the first study to find that not only the extraction kinetic but also the degradation kinetic of rutin is well described by the two-site kinetic model in noni fruit using SW.

Most of the studies carried out in subcritical water extraction highlight fast and slow extraction behaviors [26,27,29,43]. Shotipruk et al. [24] examined pressurized hot water extraction of anthraquinones from dried roots of noni, and the extraction kinetic analysis suggested that the extraction process did not correspond strictly to any one of the cases, such as the intraparticle diffusion or thermodynamic partition. Anekpankul et al. [25] also investigated subcritical water extraction of damnacanthal from the dried root of noni, and the extraction was controlled by a combination of the intraparticle diffusion and thermodynamic partition, and the extraction behavior may vary depending on extraction conditions.

### 3.4. Diffusion Coefficient and Activation Energy

The mass transfer properties of phenolic compounds from noni fruits using SW were determined by the diffusion coefficient (*D_e_*) using the equation suggested by other authors [23,44]. The *D_e_* value of scopoletin was only 0.51 × 10^−9^ m^2^/s at 100 °C, 1 mL/min, but increased to 1.93 × 10^−9^ m^2^/s (3.7-fold) at 140 °C, 3 mL/min (Table 3). The *D_e_* value of alizarin also increased 16.2-fold with increasing extraction temperature and flow rate from 100 °C, 1 mL/min (0.09 × 10^−9^ m^2^/s), to 140 °C, 3 mL/min (1.46 × 10^−9^ m^2^/s). The *D*_e_ values of rutin cannot be determined due to the thermal degradation at 140 °C.

The increases in *D_e_* value with increasing flow rate and temperature may be due to the increase in mobility of the solute at a high temperature [43] and the increase in the concentration gradient of the solute between the solvent and the sample [45,46]. Kim and Lim [44] also reported that the *D_e_* values of hesperidin and polymethoxyflavones from *Citrus unshiu* peel using SW increased from 0.13 × 10^−9^ and 0.42 × 10^−9^ cm^2^/s to 1.28 × 10^−9^ and 1.62 × 10^−9^ cm^2^/s, respectively, as the increase in temperature and flow rate from 120 °C, 1 mL/min, to 160 °C, 2 mL/min.

The activation energies (*E_a_*) for diffusion of scopoletin and alizarin at various flow rates were determined using the Arrhenius-type equation suggested by other authors [30,44]. The *E_a_* values of alizarin at all flow rates (62.7–64.5 kJ/mol) were 2.9- to 8.5-fold higher than those of scopoletin (7.3–22.0 kJ/mol) at different flow rates (Table 4), probably because the solubility of neat alizarin is low in water [47]. Alizarin is almost insoluble in water, but readily soluble in alcohol and alkaline solutions [35]. The *E_a_* values of scopoletin increased from 7.3 kJ/mol at 1 mL/min to 22.0 kJ/mol at 3 mL/min, while those of alizarin did not vary depending on the flow rate. This difference is probably due to the molecular structure or existing form in the sample matrix (scopoletin: free (aglycone), alizarin: bound (glycoside)). Kim and Lim [44] also reported that the *E_a_* value of polymethoxyflavones (aglycone) increased from 7.1 to 25.8 kJ/mol, while that of hesperidin (glycoside) decreased from 42.3 to 37.2 kJ/mol with an increase in water flow rate from 1 to 2 mL/min.

## 4. Conclusions

Bioactive phenolic compounds were extracted from noni fruits using a custom-made semicontinuous SWE system. The highest yields of scopoletin (530.6 µg/g dry sample (99.4% compared with the content in the raw sample)) and alizarin (81.5 µg/g dry sample (348%)) were obtained at 140 °C, 3 mL/min, while that of rutin (476.2 µg/g dry sample (87.3%)) was at 120 °C, 3 mL/min. The two-site kinetic desorption model best describes the extraction kinetics of scopoletin, rutin, and alizarin from noni fruits under all extraction conditions using SW. The diffusion coefficients of scopoletin and alizarin increased about 3.78- and 16.2-fold at 140 °C relative to 100 °C. The activation energy of scopoletin increased, while that of alizarin did not vary with an increase in the flow rate. The extraction kinetics revealed in this study could be useful to provide useful information for process design at the industrial level of subcritical water extraction devices. Further works are needed on the analysis of free (aglycone) and bound (glycoside) alizarins in the raw sample and the extracts.

## Figures and Tables

**Table 1:** The phenolic compound yields from noni fruits by subcritical water extraction at different temperatures and flow rates.

No.	Temperature (°C)	Flow Rate (mL/min)	Extraction Yield (µg/g Dry Sample)		
			Scopoletin	Alizarin	Rutin
1	100	1	364.2 ± 11.7	15.0 ± 0.8	372.9 ± 11.7
2		2	435.3 ± 6.4	19.1 ± 0.3	429.6 ± 11.9
3		3	444.8 ± 3.3	20.0 ± 0.6	437.3 ± 4.8
4	120	1	382.1 ± 9.5	28.8 ± 1.1	375.5 ± 12.0
5		2	441.3 ± 4.9	34.5 ± 2.3	457.0 ± 15.7
6		3	492.6 ± 11.9	34.7 ± 1.8	476.2 ± 16.6
7	140	1	416.4 ± 3.2	64.0 ± 1.0	368.8 ± 2.6
8		2	493.3 ± 11.5	78.3 ± 2.8	385.2 ± 12.1
9		3	530.6 ± 22.1	81.5 ± 0.8	452.0 ± 16.2

Data are mean ± SD (*n* = 3).

**Table 2:** Partitioning coefficients (K_D_) of bioactive compounds extracted by subcritical water fitted by the thermodynamic partitioning model.

Temperature (°C)	Flow Rate (mL/min)	Partitioning Coefficients (K_D_)		
		Scopoletin	Alizarin	Rutin
100	1	9.0 ± 0.6 ^a^	71.6 ± 2.7 ^a^	9.0 ± 0.5 ^a^
	2	9.1 ± 0.5 ^a^	70.2 ± 1.9 ^a^	9.2 ± 0.4 ^a^
	3	9.3 ± 0.6 ^a^	74.2 ± 4.5 ^a^	9.3 ± 0.4 ^a^
120	1	8.1 ± 0.4 ^b^	51.2 ± 2.5 ^b^	6.7 ± 0.2 ^c^
	2	8.1 ± 0.4 ^b^	52.5 ± 2.4 ^b^	6.5 ± 0.4 ^c^
	3	7.5 ± 0.5 ^bc^	53.9 ± 2.5 ^b^	6.7 ± 0.2 ^c^
140	1	7.2 ± 0.1 ^c^	9.6 ± 0.3 ^c^	7.8 ± 0.1 ^b^
	2	7.5 ± 0.3 ^bc^	9.2 ± 0.8 ^c^	7.9 ± 0.6 ^b^
	3	7.7 ± 0.0 ^bc^	9.1 ± 0.5 ^c^	7.5 ± 0.3 ^b^

Data are mean ± SD (*n* = 3). Mean values with different characters in each column are significantly different (*p* < 0.05 by Duncan’s multiple range test).

**Table 3:** Diffusion coefficients of scopoletin and alizarin at various water temperatures and flow rates.

Temperature (°C)	Flow Rate (mL/min)	Diffusion Coefficient (×10^−9^ m^2^/s)	
		Scopoletin	Alizarin
100	1	0.51 ± 0.03 ^f^	0.09 ± 0.01 ^f^
	2	0.71 ± 0.03 ^de^	0.14 ± 0.00 ^ef^
	3	0.96 ± 0.02 ^c^	0.19 ± 0.01 ^de^
120	1	0.55 ± 0.02 ^ef^	0.18 ± 0.01 ^de^
	2	0.86 ± 0.02 ^cd^	0.22 ± 0.02 ^d^
	3	1.20 ± 0.03 ^b^	0.25 ± 0.03 ^d^
140	1	0.64 ± 0.01 ^ef^	0.61 ± 0.01 ^c^
	2	1.14 ± 0.13 ^b^	1.02 ± 0.07 ^b^
	3	1.93 ± 0.26 ^a^	1.46 ± 0.11 ^a^

Data are mean ± SD (*n* = 3). Mean values with different characters in each column are significantly different (*p* < 0.05 by Duncan’s multiple range test).

**Table 4:** Activation energies of scopoletin and alizarin during subcritical water extraction.

Flow Rate (mL/min)	Activation Energy (kJ/mol)	
	Scopoletin	Alizarin
1	7.3 ± 1.3 ^b^	62.7 ± 2.0 ^a^
2	15.0 ± 4.9 ^ab^	63.6 ± 1.8 ^a^
3	22.0 ± 4.6 ^a^	64.5 ± 1.8 ^a^

Data are mean ± SD (*n* = 3). Mean values with different characters in each column are significantly different (*p* < 0.05 by Duncan’s test).

## Data Availability

Data are not shared.

## Supplementary Materials

The following are available online at https://www.mdpi.com/article/10.3390/foods10102260/s1: Figure S1: HPLC chromatograms of standard scopoletin and rutin at 350 nm and alizarin at 250 nm, Figure S2: HPLC chromatograms of methanol extract from noni fruits: scopoletin (RT: 9.85) and rutin (RT: 11.36) at 350 nm (a), and alizarin (RT: 24.53) at 250 nm (b), Figure S3: HPLC chromatograms of subcritical water extract at 140 ℃ and 1 mL/min from noni fruits: scopoletin (RT: 9.87) and rutin (RT: 11.39) at 350 nm (a), and alizarin (RT: 24.54) at 250 nm (b), Table S1: The values of *f*, *k_1_*, and *k_2_* of bioactive compounds extracted by subcritical water fitted by the two-site kinetic model, Table S2: Determination coefficients and root mean square error values for the thermodynamic partitioning and two-site kinetic models.
