# Supplementary material for: Kinetic Study of Subcritical Water Extraction of Scopoletin, Alizarin, and Rutin from Morinda citrifolia
Source: Foods. 2021 Sep 24;10(10):2260. doi: 10.3390/foods10102260 (PMC8534878; doi:10.3390/foods10102260)
Supplement: Supplementary file 1 [file foods-10-02260-s001.zip › foods-1387402-supplementary.pdf]

Supplementary materials

Diffusion Coefficient (D_e, m^2/s)

$$1 - \frac{C}{C_0} = \frac{6}{\pi^2}\exp(-\frac{D_e\pi^2 t}{r^2})$$

where C and C_0 are the solute mass (mg/g) extracted after time, t (min) and in the raw sample, respectively, and r is the particle radius (mm).

Activation Energy (E_a, kJ/mol)

$$D_e = D_0\exp(-\frac{E_a}{RT})$$

where D_e and D_o are the effective and initial diffusion coefficients (m^2/s), respectively, T is the absolute temperature (K), and R is the molar gas constant (J·K/mol).

(a) Scopoletin 15 mg/L

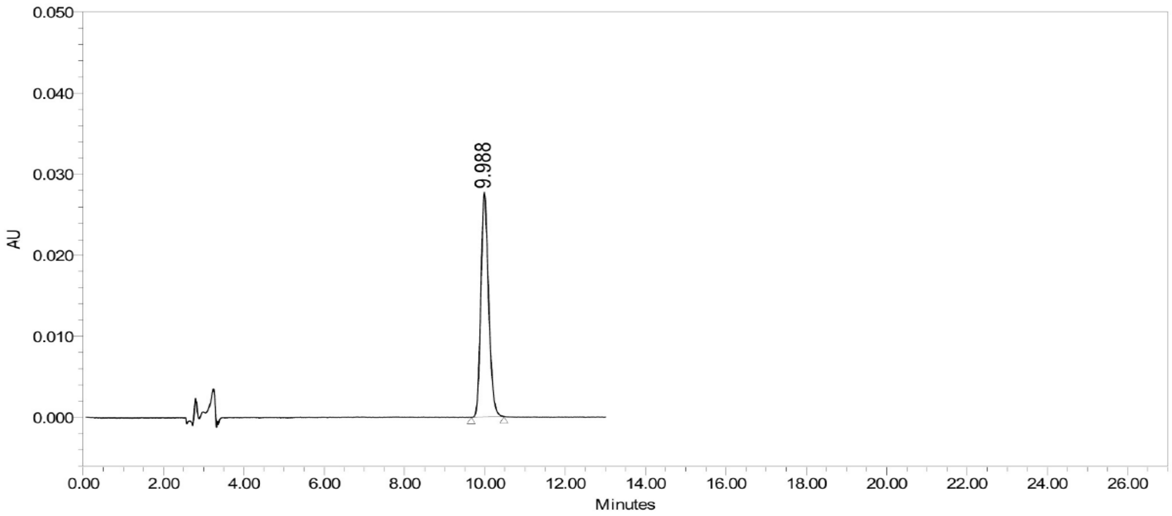

(b) Rutin 15 mg/L

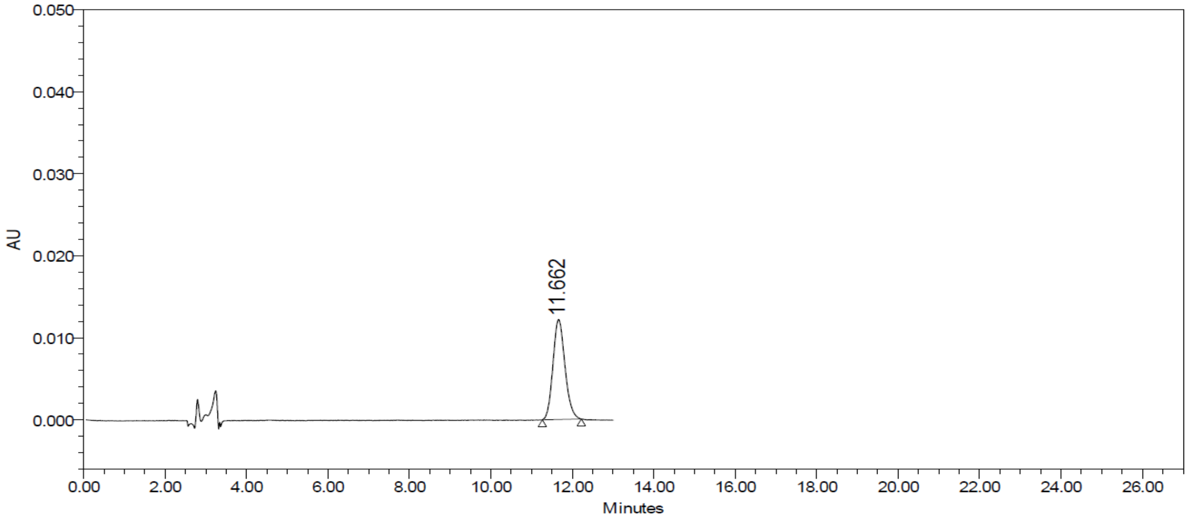

(c) Alizarin 1.2 mg/L

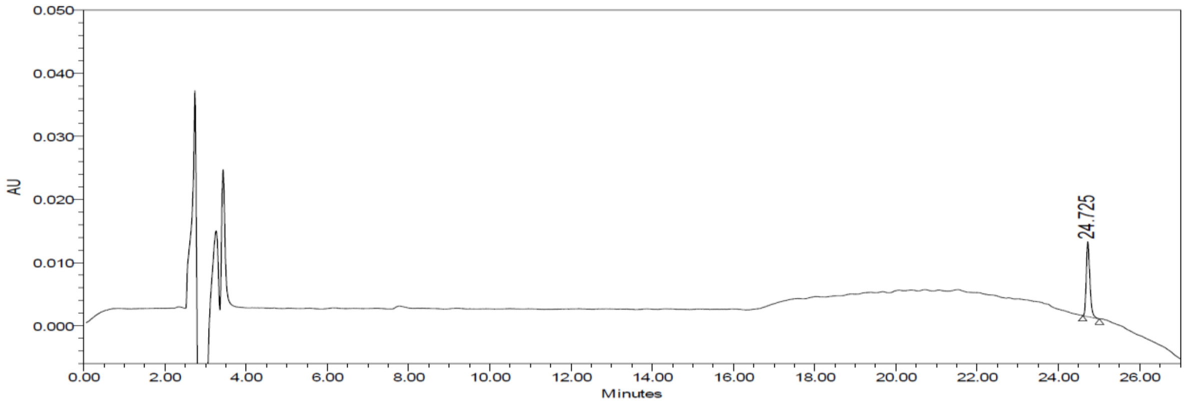

Figure S1. HPLC chromatograms of standard scopoletin and rutin at 350 nm and alizarin at 250 nm.

(a) 350 nm

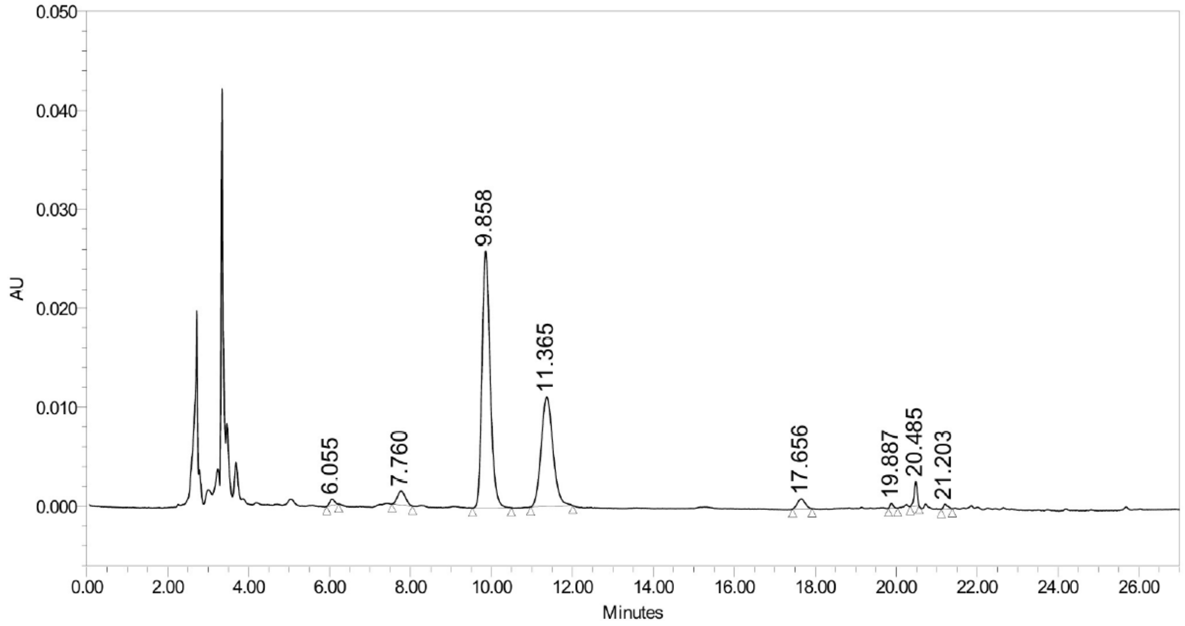

(b) 250 nm

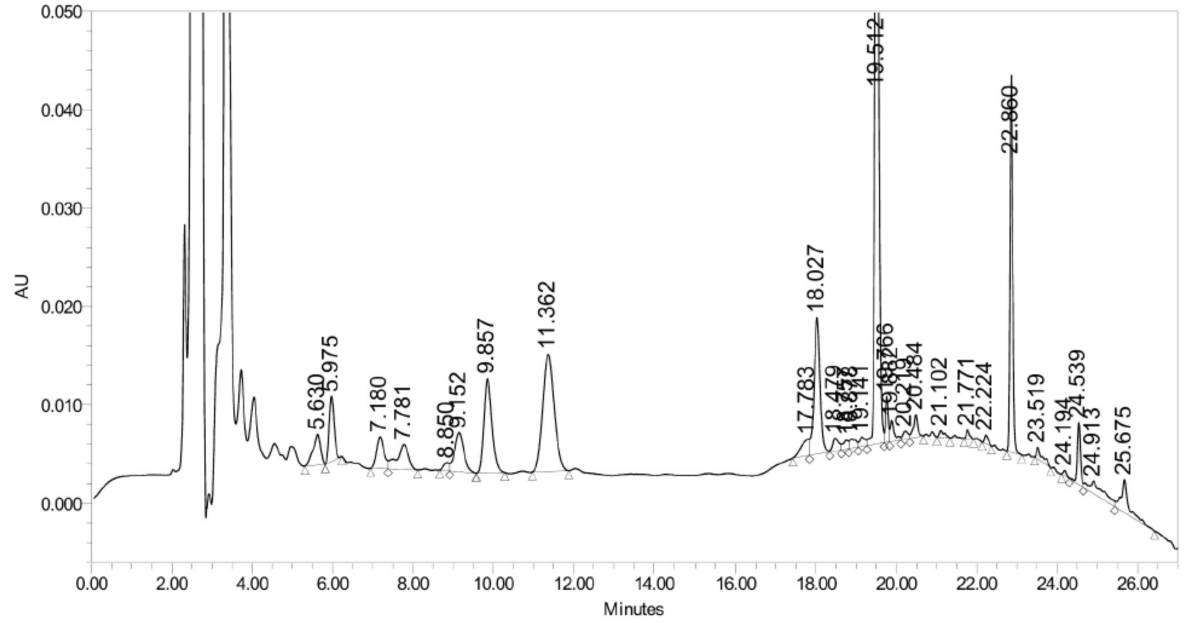

Figure S2. HPLC chromatograms of methanol extract from noni fruits; scopoletin (RT: 9.85) and rutin (RT: 11.36) at 350 nm (a), and alizarin (RT: 24.53) at 250 nm (b).

(a) 350 nm

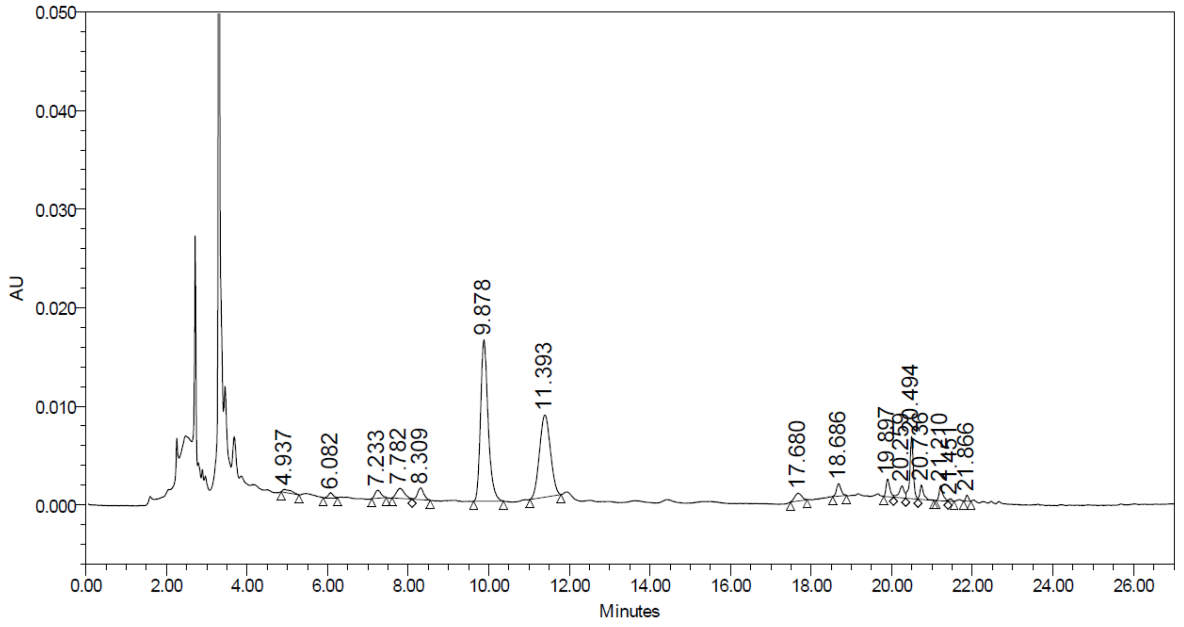

(b) 250 nm

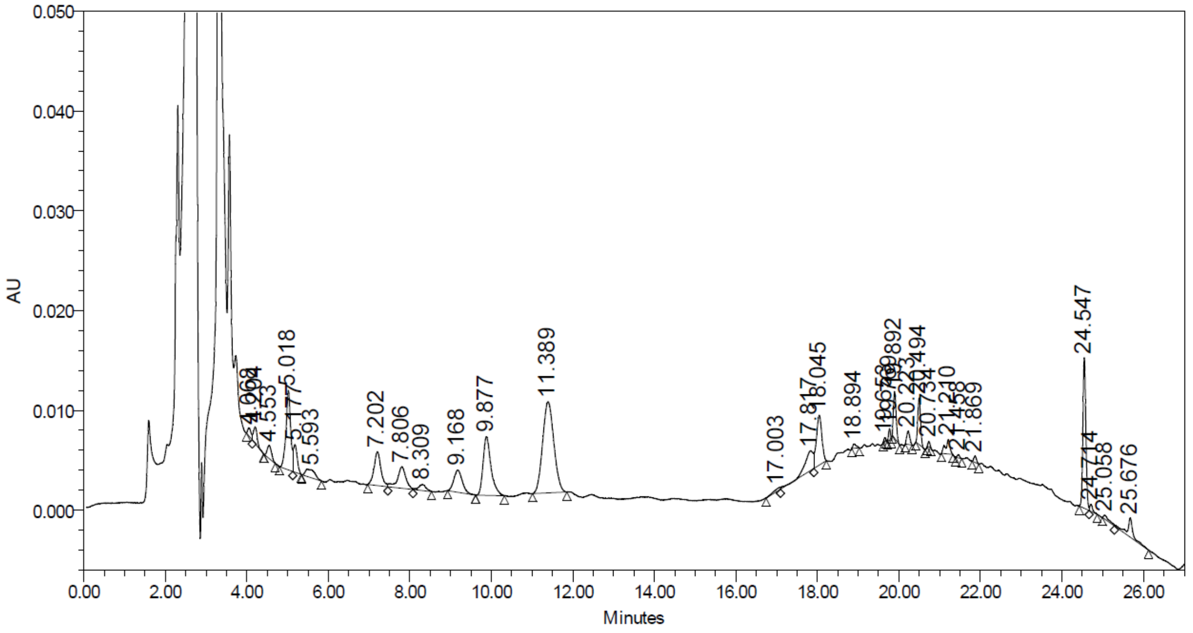

Figure S3. HPLC chromatograms of subcritical water extract at 140 ℃ and 1 mL/min from noni fruits; scopoletin (RT: 9.87) and rutin (RT: 11.39) at 350 nm (a), and alizarin (RT: 24.54) at 250 nm (b).

Table S1. The values of f, k_1, and k_2 of bioactive compounds extracted by subcritical water fitted by the two-site kinetic desorption model.

Temperature (°C)	Flow rate (mL/min)	Scopoletin			Alizarin			Rutin		
		f	k_1	k_2	f	k_1	k_2	f	k_1	k_2
100	1	0.5134 ± 0.0100^{e}	0.0571 ± 0.0035^{f}	0.0571 ± 0.0011^{c}	0.2248 ± 0.0131^{f}	0.0108 ± 0.0007^{g}	0.0093 ± 0.0006^{d}	0.5815 ± 0.0249^{c}	0.0674 ± 0.0023^{g}	0.0406 ± 0.0020^{b}
	2	0.7129 ± 0.0103^{d}	0.1263 ± 0.0051^{d}	0.0421 ± 0.0021^{d}	0.2937 ± 0.0036^{e}	0.0749 ± 0.0024^{de}	0.0012 ± 0.0001^{e}	0.8503 ± 0.0376^{a}	0.1234 ± 0.0039^{f}	0.0044 ± 0.0002^{c}
	3	0.8536 ± 0.0181^{c}	0.1864 ± 0.0076^{b}	0.0095 ± 0.0002^{f}	0.2459 ± 0.0123^{f}	0.1453 ± 0.0080^{b}	0.0011 ± 0.0001^{e}	0.7881 ± 0.0376^{b}	0.1871 ± 0.0114^{c}	0.0035 ± 0.0002^{c}
120	1	0.5348 ± 0.0098^{e}	0.0621 ± 0.0030^{ef}	0.0620 ± 0.0031^{b}	0.2948 ± 0.0120^{e}	0.0207 ± 0.0011^{g}	0.0207 ± 0.0011^{b}	0.5951 ± 0.0274^{c}	0.0780 ± 0.0024^{g}	0.0430 ± 0.0024^{ab}
	2	0.7144 ± 0.0339^{d}	0.1438 ± 0.0044^{c}	0.0419 ± 0.0028^{d}	0.4173 ± 0.0168^{d}	0.0581 ± 0.0086^{f}	0.0142 ± 0.0032^{c}	0.8611 ± 0.0270^{a}	0.1702 ± 0.0069^{d}	0.0055 ± 0.0007^{c}
	3	0.8754 ±	0.1763 ±	0.0389 ±	0.4216 ±	0.0838 ±	0.0070 ±	0.8506 ±	0.2000 ±	0.0037 ±

		0.0170[bc]	0.0031[b]	0.0017[d]	0.0019[d]	0.0222[d]	0.0018[d]	0.0220[a]	0.0073[b]	0.0003[c]
140	1	0.5382 ±	0.0704 ±	0.0734 ±	0.5403 ±	0.0651 ±	0.0651 ±	0.5509 ±	0.0705 ±	0.0443 ±
		0.014[e]	0.0014[e]	0.0014[a]	0.0147[c]	0.0005[ef]	0.0005[a]	0.0251[c]	0.0027[g]	0.0030[a]
	2	0.9011 ±	0.1519 ±	0.0606 ±	0.7780 ±	0.1163 ±	0.0627 ±	0.7622 ±	0.1375 ±	0.0010 ±
		0.0290[b]	0.0069[c]	0.0015[b]	0.0236[b]	0.0086[c]	0.0063[a]	0.0172[b]	0.0056[e]	0.0002[d]
	3	0.9925 ±	0.2220 ±	0.0279 ±	0.8817 ±	0.1986 ±	0.0642 ±	0.8370 ±	0.2401 ±	0.0008 ±
		0.0030[a]	0.0143[a]	0.0004[e]	0.0281[a]	0.0025[a]	0.0038[a]	0.0307[a]	0.0068[a]	0.0000[d]

Data are the mean ± SD (n = 3). The mean values with different letters in each column are significantly different ($p < 0.05$ by Duncan test).

Table S2. Determination coefficients (R^2) and root mean square error values (RMSE) for the thermodynamic partitioning and two-site kinetic desorption models.

Temperature (°C)	Flow rate (ml min^{-1})	Scopoletin				Alizarin				Rutin			
		K_D		Two-site		K_D		Two-site		K_D		Two-site	
		R^2	RMSE (×10^2)	R^2	RMSE (×10^2)	R^2	RMSE (×10^2)	R^2	RMSE (×10^2)	R^2	RMSE (×10^2)	R^2	RMSE (×10^2)
100	1	0.9926	1.99	0.9917	2.34	0.9940	0.74	0.9943	0.63	0.9979	1.19	0.9970	2.90
	2	0.9990	1.31	0.9993	0.69	0.9900	1.13	0.9976	0.99	0.9984	2.08	0.9997	0.54
	3	0.9993	3.40	0.9999	2.31	0.9671	2.48	0.9967	0.50	0.9897	4.50	0.9988	1.76
120	1	0.9962	1.48	0.9946	1.97	0.9903	2.40	0.9962	0.83	0.9960	2.09	0.9973	1.18
	2	0.9994	1.60	0.9990	0.83	0.9924	1.78	0.9994	1.45	0.9990	2.64	0.9978	2.41
	3	0.9969	2.10	0.9997	0.50	0.9929	1.47	0.9998	0.36	0.9970	4.18	0.9969	1.54
140	1	0.9995	1.04	0.9960	1.91	0.9991	2.23	0.9929	2.52	0.9997	0.86	0.9997	0.37
	2	0.9957	2.41	0.9987	1.10	0.9950	2.81	0.9953	2.35	0.9997	4.78	0.9993	0.66
	3	0.9876	4.56	0.9964	2.08	0.9954	2.72	0.9955	2.19	0.9862	4.76	1.0000	0.16
